# Binding Modes of a Phenylpyridinium Styryl Fluorescent Dye with Cucurbiturils

**DOI:** 10.3390/molecules25215111

**Published:** 2020-11-03

**Authors:** Adrien Paudics, Dóra Hessz, Márton Bojtár, Benjámin Gyarmati, András Szilágyi, Mihály Kállay, István Bitter, Miklós Kubinyi

**Affiliations:** 1 Department of Physical Chemistry and Materials Science, Budapest University of Technology and Economics, 1521 Budapest, Hungary; paudics.adrien@mail.bme.hu (A.P.); hessz.dora@mail.bme.hu (D.H.); bgyarmati@mail.bme.hu (B.G.); aszilagyi@mail.bme.hu (A.S.); kallay@mail.bme.hu (M.K.); 2 “Lendület” Chemical Biology Research Group, Institute of Organic Chemistry, Research Centre for Natural Sciences, 1519 Budapest, Hungary; bojtar.marton@ttk.hu; 3 Department of Organic Chemistry and Technology, Budapest University of Technology and Economics, 1521 Budapest, Hungary; ibitter@mail.bme.hu

**Keywords:** molecular recognition, host–guest complex, hydrophobic interactions, size selectivity

## Abstract

In order to explore how cucurbituril hosts accommodate an *N*-phenyl-pyridinium derivative guest, the complexation of the solvatochromic dye, 4-(4-(dimethylamino)styryl)-1-phenylpyridinium iodide (PhSt) with *α,α′,δ,δ′*-tetramethyl-cucurbit[6]uril (Me_4_CB6) and cucurbit[7]uril (CB7) was investigated by absorption spectroscopic, fluorescence and NMR experiments. In aqueous solutions, PhSt forms 1:1 complexes with both cucurbiturils, the complex with CB7 has a higher stability constant (*K_a_* = 6.0 × 10^6^ M^−1^) than the complex with Me_4_CB6 (*K_a_* = 1.1 × 10^6^ M^−1^). As revealed by NMR experiments and confirmed by theoretical calculations, CB7 encapsulates the whole phenylpyridinium entity of the PhSt cation guest, whereas the cavity of Me_4_CB6 includes only the phenyl ring, the pyridinium ring is bound to the carbonyl rim of the host. The binding of PhSt to cucurbiturils is accompanied by a strong enhancement of the fluorescence quantum yield due to the blocking of the deactivation through a twisted intramolecular charge transfer (TICT) state. The TICT mechanism in PhSt was characterized by fluorescence experiments in polyethylene glycol (PEG) solvents of different viscosities. The PhSt-CB7 system was tested as a fluorescence indicator displacement (FID) assay, and it recognized trimethyl-lysine selectively over other lysine derivatives.

## 1. Introduction

Cucurbiturils (CB-s), with their electron-rich carbonyl portals and hydrophobic cavities, are particularly suitable hosts for the binding of organic cations [1]. Quaternary pyridinium derivatives are one class of cationic species, the binding of which to CB-s has been studied extensively. In the CB6 (cucurbit[n]urils are denoted as CBn-s) complexes of alkylpyridinium cations, only the alkyl chain intrudes into the host cavity; the charged N interacts with the electron-rich CO groups of the CB portal [2,3,4]. In similarity, CB7 includes only the alkyl chain of 4-naphthyl-*N*-alkylpyridinium cation [5] and one of the alkyl chains of dialkyl-4,4′-bipyridiniums (viologens) with longer alkyl chains, but it also accommodates one of the pyridinium nuclei of dimethyl and diethyl viologens [6]. CB7 encapsulates only the pyridinium ring of the derivative in which a bulky anthracene substituent is linked to the ring N atom via an alkyloxy bridge, whereas CB8 fully encapsulates this large guest [7]. Furthermore, CB8 can bind a pair of stacked *N*-benzylpyridinium [8] or 4-aryl-*N*-methylpyridinium derivative guests in its cavity [9]. Studies on the complexes of 4-pyrrolidino-*N*-alkylpyridinium cations with CB7 and CB8 concluded that the CB hosts encapsulate such guests completely or shuttle on them, depending on the length of the alkyl chain of the guest and the cavity size of the host [10,11].

The positive charge of cationic guests is shielded in CB complexes which is a mean to modify the properties of pyridinium derivatives, like the reduction potential [12] or in the case of guests with protonable functional groups, the pKa [13]. The CB complexes of environment-sensitive fluorescent dyes receive much attention, in particular, because many of these complexes function as molecular optical sensors for the detection of non-fluorescent chemical species [14,15]. The phenyl pyridinium dye, MeSt is one example for the water-soluble indicators, the spectral properties of which can greatly change on complexation by cucurbiturils [13,16,17] and also by other macrocyclic receptors (cyclodextrins [18], sulfonatocalixarenes [19], and carboxylato-pillararenes [20,21]). Exploiting these effects, displacement assays have been constructed with MeSt as an indicator, for the fluorescence detection of non-fluorescent analytes [19,20,21,22,23].

**Figure 1:**
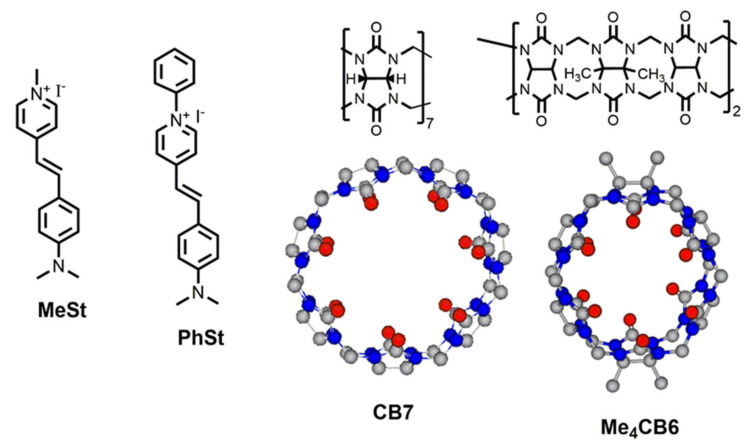
Structures of the indicator dyes MeSt and PhSt and cucurbiturils CB7 and Me_4_CB6.

In the present work, we studied the complexation of the styryl pyridinium dye PhSt with cucurbiturils Me_4_CB6 and CB7 (see Figure 1) and demonstrated that the system CB7-PhSt functions as a displacement assay which discriminates lysine and its methylated derivatives. So far, the PF_6_^−^ salt of PhSt cation attracted research attention, which showed strong nonlinear optical properties [24,25,26]. As far as we know, supramolecular complexes of PhSt with CB-s have not been described yet, only its complex with a pillararene macrocycle was discussed briefly in our earlier work [27]. We chose Me_4_CB6 instead of CB6 because the tetramethyl derivative dissolves in water much better [28]. The water solubility of CB7 is satisfactory [29].

Although the structures of a number of various complexes between quaternary pyridinium cations and CB-s have been reported, to our knowledge, these studies did not extend to the complexes of *N*-phenyl-pyridinium derivatives. The aims of our work were (i) to describe the structures of the complexes of PhSt with the six- and seven-membered CB macrocyles, (ii) to explore the changes in the optical spectra of PhSt upon the complexation with CB-s, (iii) to construct a CB7-PhSt assay, which we hoped would display similarly advantageous spectroscopic properties as the CB6-MeSt assay [22], but capable of detecting biomolecules larger than aliphatic diamines, like many CB7-based assays reported in the literature [30].

Prior to the experiments on complexation, the excited state properties of uncomplexed PhSt were characterized by spectral measurements in solvents of different polarities and viscosities.

## 2. Results and Discussion

### 2.1. Scheme of Photoinduced Processes

**Figure 2:**
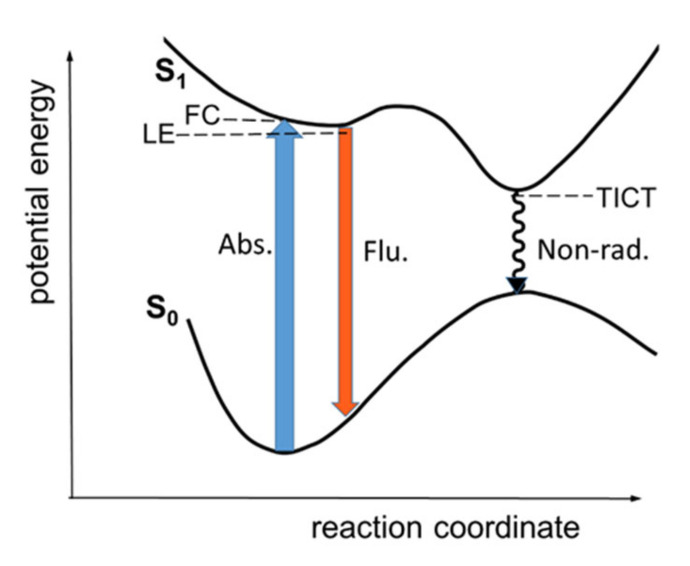
Schematic one-dimensional S_0_ and S_1_ state potential energy curves for PhSt.

The photophysical properties of PhSt were interpreted in terms of the scheme of photoinduced processes in Figure 2, which is similar to the scheme proposed for the photocycle of MeSt on the basis of detailed experimental and theoretical studies [31,32,33]. The model considers two routes for the deexcitation. Following the photoexcitation into the Franck-Condon (FC) state, the dye returns to the S_0_ surface either from a partially relaxed ‘locally excited’ (LE) state, which a radiative process, or the relaxation goes through a TICT state which is dark. The latter is considered the dominant non-radiative process. In MeSt, the rotation around the aniline-vinylene bond is the likely TICT process [32,34,35,36].

### 2.2. Solvatochromism

**Figure 3:**
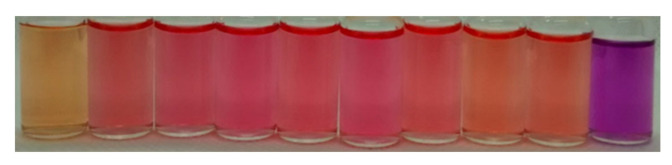
Photograph of 2 × 10^−5^ M solutions of PhSt in (from left to right) water, methanol, ethanol, *n*-propanol, *i*-propanol, *n*-butanol, *t*-butanol, acetonitrile, acetone, and dichloromethane.

As can be seen in Figure 3, PhSt shows a spectacular solvatochromism. The absorption and fluorescence spectra of PhSt in seven protic and three aprotic solvents are displayed in Figure S1 in the Supplementary Materials (SM), the spectral data are collected in Table 1. Comparing the band positions of PhSt to the band positions of MeSt in identical solvents (for the spectral data of MeSt see Ref. [37]), the absorption band of PhSt appears at about 30 nm longer wavelengths, its fluorescence band at 60–80 nm longer wavelengths. The location of the fluorescence in the red range and the enhanced Stokes shift are beneficial features of PhSt as a fluorescence probe.

**Figure 4:**
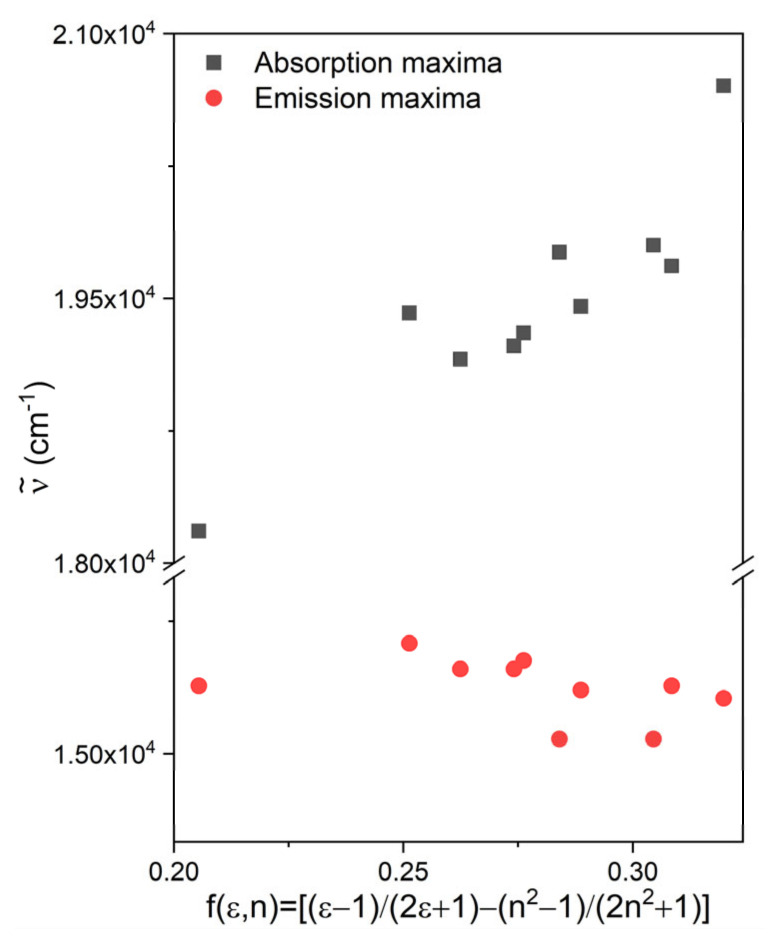
Plot of the wave numbers of the charge transfer (CT) transition of PhSt vs. solvent polarity function.

The wave numbers of the absorption and fluorescence bands of PhSt are plotted as functions of the solvent polarity function (orientation polarizability), *f*(*ε,n*) in Figure 4. As these diagrams show, the absorption maximum shifts to the blue with increasing solvent polarity, i.e., PhSt is a negatively solvatochromic dye. The position of the fluorescence band is less sensitive to the solvent. Overall, the dye shows a weak positive fluorosolvatochromism. The spectral data measured in CH_2_Cl_2_ deviate from the above trends, which may suggest that PhSt forms loose ion pairs in solvents of low polarity [38].

These trends are similar to the trends observed in the spectra of MeSt and their quantitative interpretation requires the consideration of both the general and specific solute-solvent interactions in the ground state and in the Frank-Condon and the relaxed excited states. Such a thorough analysis involves sophisticated time-resolved spectroscopic experiments and quantum chemical calculations like those performed for the 2-pyridinium isomer of MeSt [39,40].

A possible qualitative explanation for the opposite solvatochromic trends in the absorption and fluorescence spectra is obtained by decomposing the absorption transition energy of the solvated dye cation as (Equation (1)):

(1) $$hv_{abs}=E_{S1}^{eq}-E_{S0}^{eq}+\Lambda_{S1}$$

and its emission transition energy as (Equation (2)):

(2) $$hv_{fl}=E_{S1}^{eq}-E_{S0}^{eq}-\Lambda_{S0}$$

where *E*_*S*0_^*eq*^ and *E*_*S*1_^*eq*^ are the energies of the cations with relaxed solvent shells, Λ_S1_ and Λ_*S*0_ are the solvent reorganization energies in the respective states [41]. Λ_*S*0_ and Λ_*S*1_ increase always with increasing solvent polarity, and in protic solvents these changes may be dominant over the change of the adiabatic transition energy, *E*_*S*1_^*eq*^ − *E*_*S*0_^*eq*^. As a result, ν*_abs_* will increase, ν*_fl_* will decrease with growing solvent polarity.

### 2.3. Viscosity Dependence of Fluorescence

**Figure 5:**
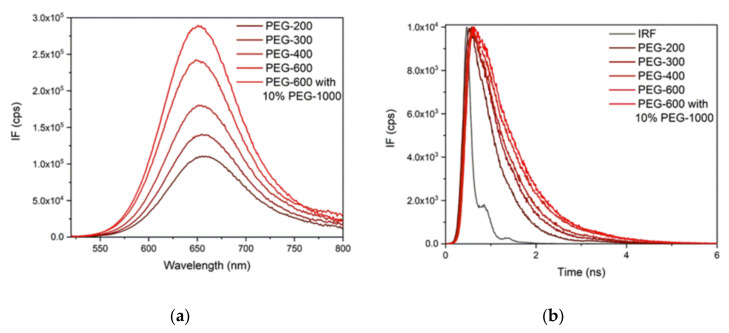
(**a**) Fluorescence spectra and (**b**) decay curves measured in polyethylene glycol (PEG) solutions. The spectra were measured at λ_ex_ = 512 nm; the decay curves were recorded using a 450 nm pulsed diode laser.

The effect of the viscosity on the fluorescence properties of PhSt was studied in a series of liquid polyethylene glycols (PEG-s) with different molar masses. The absorption spectra in PEG solvents did not show solvatochromism, the band maximum fell to 512 nm in all the samples (see Figure S2 in the SM). Therefore, it could be presumed that the differences in the fluorescence behavior are due to viscosity effects, the polarity effects are closely identical. The fluorescence spectra and decay curves measured in PEG solutions are displayed in Figure 5.

The fluorescence bands shifted to shorter wavelengths and their intensity increased with growing viscosity, showing that the relaxation on the S_1_ surface was slower at higher viscosities. The viscosity dependence of the fluorescence data of dyes, the excited state relaxation of which follow the scheme in Figure 2, are usually evaluated in terms of the phenomenological Equation (3):

(3) $$k_{nr}=\frac{B}{\eta^{a}}$$

where *B* and *α* are empirical parameters [42]. The rate coefficients of the radiative and non-radiative deactivations, *k_r_* and *k_nr_*, can be obtained from the fluorescence lifetime and fluorescence quantum yield, using the set of Equations (4) and (5):

(4) $$\frac{1}{\tau_{F}}=k_{F}=k_{r}+k_{nr}$$

(5) $$\Phi_{F}=\frac{k_{r}}{k_{r}+k_{nr}}$$

As shown in Table 2, the values of *k_r_* fall all in the range of 1.5–2.0 × 10^8^ s^−1^ and do not show a clear trend with the viscosity. In contrast, the values of *k_nr_* decrease monotonically with the viscosity. *k_nr_* is a total rate coefficient for all the non-radiative deactivations from the LE state, with contributions from the decay through the TICT state and the decays via a direct internal conversion and inter-system crossing. The decomposition of *k_nr_* into the contributions of the individual deactivation processes requires more exhaustive studies. Such studies have been made on the deactivation pathways in 2-DASPMI, an isomer of MeSt, which concluded that the decay via the TICT state is the dominant deactivation channel [39,43].

**Figure 6:**
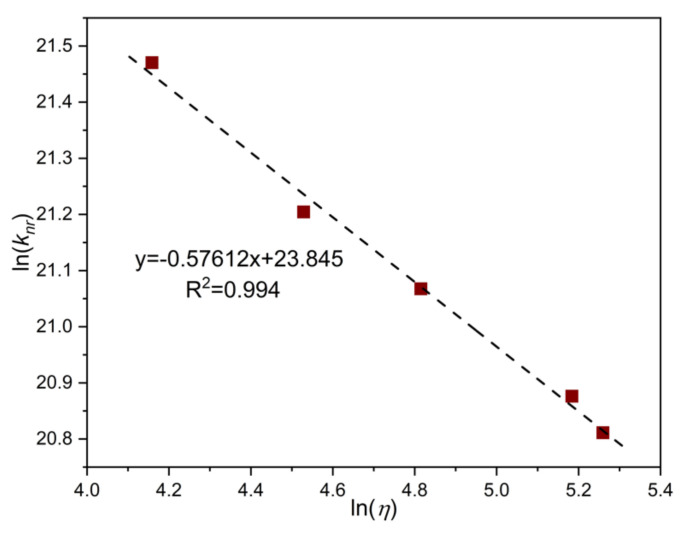
Ln-ln plot of the rate coefficients of the non-radiative decay of PhSt in PEG solutions vs. solvent viscosities.

Confirming the validity of Equation (1) for our systems, ln(*k_nr_*) correlates linearly with ln(*η*) (see Figure 6), and the slope of the fitted line is α = 0.58. This result can be compared with α = 0.94 for MeSt determined in PEG solutions [35]. The decrease of α with the mass of the substituent may indicate the coupling of the TICT process with a low-frequency motion of the Me or Ph group [35].

### 2.4. Complexation of PhSt with CB7

Preliminary absorption spectroscopic measurement in aqueous HCl and buffered solutions indicated that PhSt is bound by CB7 almost completely even at low concentrations and the complex obtained is a much stronger base than the free dye. Titrations yielded *pK*_a_ = 3.32 for PhSt and *pK*_a_ = 5.08 for PhSt⋅CB7 (see Figures S3 and S4 in the SM).

**Figure 7:**
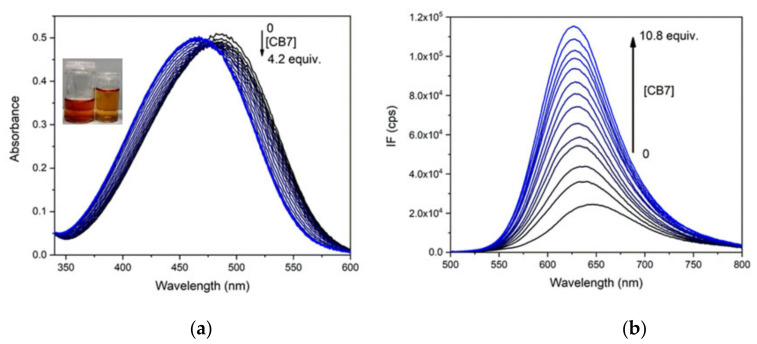
(**a**) Absorption spectra of 1.5 × 10^−5^ M solution of PhSt in pH 8.0 buffers in the presence of 0–4.2 equiv. CB7, (**b**) fluorescence spectra of 1.0 × 10^−6^ M solution of PhSt in the presence of 0–10.8 equiv. CB7, λ_ex_ = 468 nm, the isobestic point of the absorption spectra.

The optical spectra of PhSt-cucurbituril mixtures were recorded in neat water and with respect to potential applications of the complexes as FID assays in biological samples also in buffers of pH 8.0. The spectral changes in the absorption and fluorescence spectra of PhSt induced by the addition of CB7 are illustrated in Figure 7. The absorption band as well as the fluorescence band exhibits a pronounced blue shift and the fluorescence intensity is strongly enhanced. A least-square fitting to the fluorescence spectra of the mixtures yielded the binding constant of *K_a_* = 6.0 × 10^6^ M^−1^ for PhSt⋅CB7 in neat water and *K_a_* = 3.0 × 10^6^ M^−1^ in pH 8.0 buffer. The lower apparent binding constant in buffer is due to the competitive binding of buffer cations to the CB7 host [44].

The absorption maximum of PhSt⋅CB7 falls to 465 nm, its fluorescence maximum is at 625 nm in the spectra obtained by fittings to the spectra PhSt-CB7 mixtures, i.e., both bands of the complex are located at lower wavelengths than the respective band of the uncomplexed PhSt in any of the solvents tried (see Table 1). These blue shifts indicate that the stabilization energy of the complex of the ground state PhSt molecule is larger than the stabilization energy of the excited state dye. The electrostatic interactions between the positive charge on its pyridinium unit and the negative local charges on the carbonyl rim of CB7 largely contribute to the stabilization of the complex. These interactions become weaker in the complex with S_1_ state PhSt in which the positive charge of the guest is transferred largely to the dimethylaniline group.

The increased fluorescence can be due to the restriction of the TICT process of PhSt dye in the cavity of the CB7 host. There are two effects accounting for the change: (i) as a torsional motion, TICT is hindered sterically in the cavity and (ii) the dipole moment is higher in the TICT than in the vertical (directly excited) state. Therefore, the TICT is energetically unfavored in the apolar cavity.

**Figure 8:**
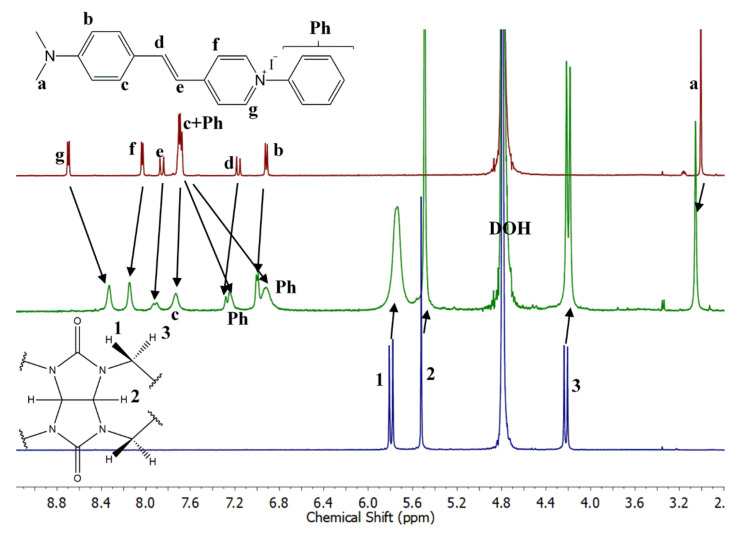
1H-NMR spectra (500 MHz, D_2_O) of (**top**) PhSt, (**middle**) PhSt-CB7 1:1 mixture, (**bottom**) CB7. [PhSt] = [CB7] = 5 × 10^−4^ M.

The NMR spectra of PhSt, CB7 and their mixture are shown in Figure 8. The assignments were confirmed by the COSY spectra displayed in Figure S7 in the SM. In the spectra of the mixtures (Figure 8) the signals of PhSt shift gradually with growing CB7 concentrations. This indicates that the exchange of the guests among the hosts are fast on the NMR timescale. The signals of the CB7 host broaden on the binding of the dye and undergo a minor upfield shift.

The spectrum of the 1:1 mixture shows a full complexation. In the spectrum of the complex, the signals of **Ph** protons shift strongly upfield, proving the encapsulation of the phenyl ring in the cucurbituril cavity. The **g** protons shift upfield, the **f** protons downfield, indicating the interaction between the positively charged pyridinium N-atom of the guest and the electron-rich carbonyl rim of the host. A similar opposite shift of the α- and β-protons of the pyridinium unit was observed in the spectrum of the CB7 complex of dibenzyl-viologen and was attributed to a similar structure with only one of the benzyl groups encapsulated [45]. The signals of the ethylene and aniline units of PhSt are shifted downfield which may derive from the reduced effective charge, i.e., the reduced electron withdrawing ability of the pyridinium group bound to the carbonyl rim of CB7.

### 2.5. Complexation of PhSt with Me_4_CB6

**Figure 9:**
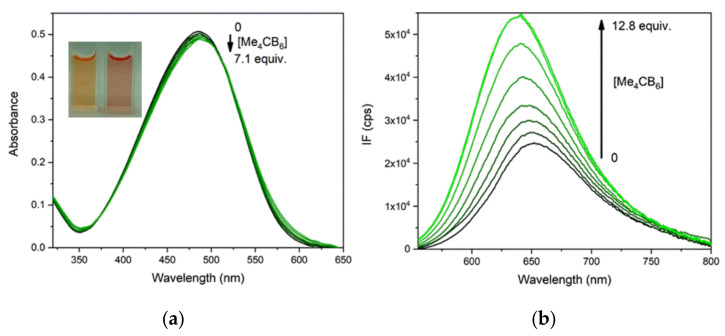
(**a**) Absorption and (**b**) fluorescence spectra of PhSt in pH 8.0 buffers in the presence of Me_4_CB6 in different concentrations. Initial concentrations of PhSt (**a**) 1.5 × 10^−5^ M. (**b**) 1.0 × 10^−6^ M, λ_ex_ = 510 nm, the isosbestic point of the absorption spectra.

On the addition of Me_4_CB6, the absorption band of PhSt shifted to the red, in contrast to the blue shift induced by CB7, while the fluorescence band of the dye showed a blue shift (see Figure 9). A fitting to the fluorescence spectra yielded *K_a_* = 1.1 × 10^6^ M^−1^ in neat water and 2.5 × 10^4^ M^−1^ in the pH 8.0 buffer. The spectral shifts seem to indicate a reduced polarity of the environment for the complex dye.

**Figure 10:**
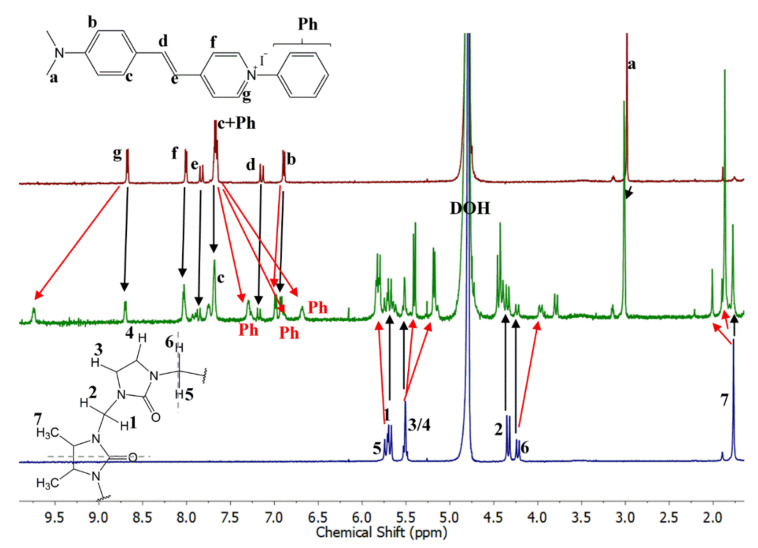
^1^H-NMR spectra (500 MHz, D_2_O) of (**top**) PhSt, (**middle**) PhSt-Me_4_CB6 1:1 mixture, (**low**) Me_4_CB6. [PhSt] = [Me_4_CB6] = 5 × 10^−4^ M.

In the NMR spectrum of the 1:1 mixture of PhSt and Me_4_CB6, the signals of the complexed and free indicators appear separately (see Figure 10). Likewise, the signals of the complexed and free host molecules also form two sets. This shows that the exchange of the guests among the hosts is slow on the NMR timescale. In the 1:1 mixture, the complexation is only partial. This is in accordance with the lower binding constant, noting that some aggregation may also affect the composition.

In contrast to the spectrum of the CB7 complex, in the spectrum of PhSt⋅Me_4_CB6, the signal of H_g_ protons shift strongly downfield, suggesting that a strong cation–π interaction acts between the pyridinium unit of the dye and the carbonyls of the CB host. A similarly strong cation–π interaction has been observed in pyridinium derivatives with covalently attached carbonyl groups [46]. In addition, the two H_g_ protons are connected to the nearest carbonyl groups of the CB7 host via hydrogen bonds, which also affects the position of their signal. The signals of the phenyl group shift upfield in a similar manner as in the spectrum of the CB7 complex, suggesting that the six-membered macrocycle also encapsulates the phenyl group of the dye. Another interesting feature of the spectrum of PhSt⋅Me_4_CB_6_ is the splitting of the signals of Me_4_CB_6_. This effect is due to the reduction of the symmetry on complexation [16].

### 2.6. Calculated Structures of the Complexes

**Figure 11:**
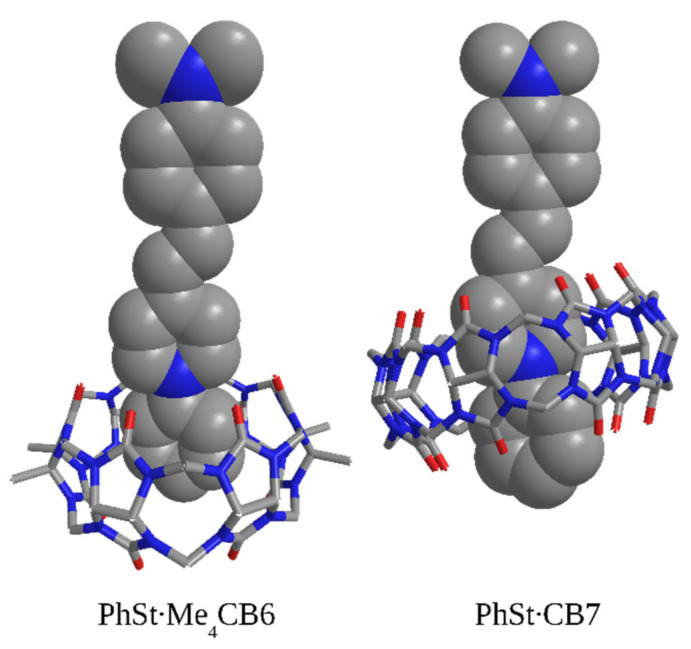
Theoretically calculated structures of the complexes PhSt-CB complexes.

The theoretical calculations identified three possible structures, both for the Me_4_CB6 and CB7 complexes of PhSt. The structures shown in Figure 11 are fully consistent with the NMR results: the Me_4_CB6 host encapsulates only the phenyl ring of PhSt in its cavity, the pyridinium N atom rests at the carbonyl rim, whereas CB7 encages the larger parts of both the phenyl and the pyridinium units of PhSt, with the pyridinium nitrogen close to the center of the cavity.

The optimized structures of the complexes in the figure suggest that there are three kinds of important interactions that stabilize the complexes. First, probably the most important interaction is the one between the positive charge located at around the pyridinium nitrogen and the carbonyl groups of the cucurbituril. Second, weak hydrogen bonds are formed between hydrogens of the pyridinium ring and the carbonyl group of the host molecules. For Me_4_CB6, this affects the hydrogen atoms located in the ortho position relative to the nitrogen, while for CB7, the hydrogens in meta position are involved. In the latter case, the interatomic distances are longer, and consequently, the interactions are weaker, but it is compensated by the interaction of the hydrogens of the phenyl ring and the carbonyl groups residing on the opposite rim of the cucurbituril ring. Third, a non-negligible amount of the binding energy is likely to stem from the dispersion interaction of the phenyl and pyridinium groups with the cucurbituril ring.

In the other two structures obtained in the calculations (Figure S8 in the Supplementary Material) the ethylene group or the phenyl ring of the dimethylaniline unit of PhSt are located within the cavities of the CB hosts. The calculated stabilization energies for all the possible structures had high values (150–188 kJ/mol, see Figure S8). A more accurate estimation of the energies would require the consideration of hydrophobic interactions.

### 2.7. Size Selectivity of Complexing

It is worth to compare the stabilities of the complexes formed by PhSt with cucurbiturils Me_4_CB6 and CB7 with the stabilities of the complexes of the reference dye MeSt with the same two cucurbituril hosts. The binding constant is 4.0 × 10^5^ M^−1^ for MeSt⋅Me_4_CB6 (in neat water) [16], whereas it is 1.35 × 10^5^ M^−1^ for MeSt⋅CB7 (in pH 7.4 buffer) [17]. Although the two values—one measured in unbuffered, the other in buffered solution—are not fully comparable, it can be said that MeSt forms complexes with Me_4_CB6 and CB7 with similar affinities. The higher stabilities of the PhSt complexes (*K_a_* = 1.1 × 10^6^ M^−1^ for PhSt⋅Me_4_CB6, *K_a_* = 6.0 × 10^6^ M^−1^ for PhSt⋅CB7 in water) originate probably from hydrophobic interactions between the phenyl group of the PhSt guest and the apolar cavity of the CB hosts. The lower stability of PhSt⋅Me_4_CB6 compared to the stability of PhSt⋅CB7 can be because the phenyl substituted pyridinium unit does not fit perfectly into the narrower rim of the six-membered macrocycle.

### 2.8. Indicator Displacement

The performance of a CB7-PhSt system as indicator displacement assay was tested with lysine (Lys), methyl lysine (LysMe), dimethyl lysine (LysMe_2_), and trimethyl lysine (LysMe_3_) as model analytes. Methods for the discrimination of lysine and methylated lysines are of key importance in biochemistry [47] since the methylation of the lysine residuals of histones is an important mechanism of epigenetic regulation of gene expression.

The displacement experiments were carried out in pH 8.0 buffer solutions. At this pH, the NMe_2_ groups of PhSt molecules are unprotonated and they remain unprotonated in the CB7 complex. (The pK_a_ of the PhStH^+^ conjugate acid is 3.32 and it shifts to 5.08 in the complex). On the basis of their pK_a_ values [48,49], the most stable structures of Lys and its methylated derivatives at pH 8.0 are those in which the head groups are in a zwitterionic state, whereas the amino end groups of Lys, LysMe and LysMe_2_ are protonated. A mixture of 10^−6^ M PhSt and 10^−6^ M CB7 was used as displacement assay in which 60% of the dye existed in complexed form.

**Figure 12:**
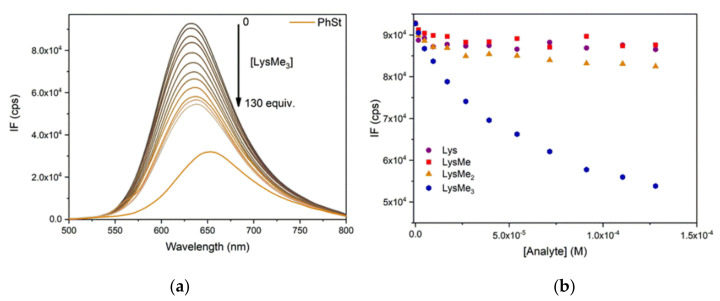
(**a)** Fluorescence spectra of PhSt-CB7 assay ([PhSt]_0_ = [CB7]_0_ = 1.0 × 10^−6^ M) in pH 8.0 buffers in the presence of 0–130 equiv. LysMe_3_; (**b**) Fluorescence intensities of the assay at 632 nm in the presence of 0–130 equivs. Lys, LysMe, LysMe_2_ and LysMe_3_. λ_ex_ = 475 nm. The spectra of PhSt-LysMe_n_-CB7 ternary systems can be seen in the Supplementary Material (Figure S9).

As shown in Figure 12, LysMe_3_ gave a much stronger turn-off signal than LysMe_2_ added in the same concentration, whereas the addition of LysMe and Lys left the fluorescence intensity almost unchanged.

The binding constants of the complexes of Lys and LysMe_n_ (*n* = 1–3) with CB7 have been determined by NMR spectroscopy in pD 4.7 deuterated buffer solutions [50]. As indicated by their pK_a_ values [48,49], the same protonated forms of lysine and methylated lysines were dominant in the weakly acidic solutions used in the NMR study as in our weakly basic solutions. The results of our indicator displacement experiments are in accordance with the order of the binding constants obtained by NMR measurements, which is K(LysMe_3_⋅CB7) > K(LysMe_2_⋅CB7) > K(LysMe⋅CB7) > K(Lys⋅CB7) [50]. This order is related to hydrophobic effects, which play a decisive role in the stabilization of CB complexes with organic guests. The hydrophobicity of the ammonium end groups of methylated lysines decreases as the methyl groups are replaced by protons, and the binding constants of the LysMe_n_⋅CB7 complexes decrease in the same direction.

## 3. Methods

### 3.1. Synthesis

The synthesis of PhSt is summarized in Scheme S1 in the SM. First, the chloride salt was prepared by the Knoevenagel condensation of 4-dimethylaminobenzaldehyde and *N*-phenylpicolinium chloride [24,27]. The chloride anion of the resulting salt was replaced with iodide using a two-step procedure through a hexafluorophosphate intermediate. Details of the synthesis are given in the SM.

Me_4_CB6 was synthesized by the method of Zhao et al. [28]. CB7, polyethylene glycols (PEG-s), lysine and methylated lysines were commercial products. The viscosities of PEG-s were measured by an Anton Paar Physica MCR 301 rheometer using a cone-plate geometry (CP25, diameter 25 mm).

### 3.2. Spectroscopic Experiments

For the absorption spectroscopic determination of the pK_a_ values, aqueous HCl solutions and Britton-Robinson buffers were used. The optical spectra of the dye—cucurbituril mixtures were measured in neat water and in Britton-Robinson buffers of pH 8.0. The fluorescence quantum yields were determined using Rh6G as a reference. The absorption spectra were recorded on an Agilent 8453 UV-VIS absorption spectrometer (Agilent Technologies, Santa Clara, CA, USA). The fluorescence spectra and decay curves were taken using an Edinburgh Instruments FS5 fluorescence spectrometer (Edinburgh Instruments, Livingston, UK). The decays were measured using an EPL 450 pulsed diode laser (pulse width 90 ps) for excitation. The spectroscopic experiments were carried out at 20 °C.

The NMR spectra were measured in D_2_O. The spectra were acquired on a 500 MHz Bruker Avance DRX-500 spectrometer (Bruker Corporation, Billerica, MA, USA).

### 3.3. Theoretical Calculations

The theoretical investigations started with the geometry optimizations of the PhSt-CB7 and PhSt-Me_4_CB6 complexes and the host and guest molecules, performed at the density functional theory (DFT) level using the ωB97X-D functional of Chai and Head-Gordon [51] and the split-valence polarized (def2-SVP) basis set of Weigend and Ahlrichs [52]. The DFT calculations were carried out with the Gaussian 09 program [53]. To calculate accurate complexation energies, local natural orbital coupled-cluster singles and doubles with perturbative triples [LNO-CCSD(T)] [54] energies were evaluated with Dunning’s augmented correlation–consistent triple-zeta (aug-cc-pVTZ) basis set [55] with the aid of the MRCC suite of quantum chemical programs [56]. In the latter calculations, the density fitting approximation was used throughout, and tight threshold settings were applied in the local correlation calculations. All calculations were performed employing the polarized continuum model (PCM) [57] with water as the solvent since the latter was employed in the experiments.

## 4. Conclusions

The binding of PhSt by cucurbiturils shows a distinct size-selectivity: the binding constant for PhSt⋅CB7 is higher than for PhSt⋅Me_4_CB6. This differs from the complexation behavior of MeSt, the CB7, and Me_4_CB6 complexes of which have close binding constants of 10^5^ M^−1^ magnitudes. The main structural difference between the complexes of PhSt with the two cucurbiturils is the different position of the pyridinium unit of the dye guest related to the cavity of the host: in the PhSt⋅CB7 complex, the pyridinium and the phenyl ring are located within the cavity. Therefore cation–pi interactions dominate, whereas in PhSt⋅Me_4_CB6 only the phenyl ring fits into the cavity, the pyridinium ring interacts with the CO portal via ion–dipole interactions. The binding of PhSt to CB7 is accompanied by a strong blue shift of the fluorescence band and a significant enhancement of the fluorescence intensity. These features make the CB7-PhSt system promising as a FID assay, capable of discriminating structurally similar biomolecules with different spacious requirements or different hydrophobicity, like lysine and its methylated derivatives.

## Figures and Tables

**Table 1:** Absorption and fluorescence spectral data of PhSt in different solvents.

Solvent	*f*(*ε,n*)	λ_abs /_ nm (ε/M^−1^cm^−1^)	λ_F_ / nm (IF/100 cps)
water	0.3198	483 (30300)	653 (75)
methanol	0.3085	508 (58300)	650 (388)
ethanol	0.2887	514 (44200)	651 (758)
1-propanol	0.2741	520 (43800)	646 (1530)
2-propanol	0.2762	518 (40800)	644 (1530)
1-butanol	0.2624	522 (46800)	646 (2480)
t-butanol	0.2513	515 (38500)	640 (2880)
acetonitrile	0.3045	505 (43700)	663 (151)
acetone	0.2840	506 (43100)	663 (228)
dichloromethane	0.2054	550 (61900)	650 (4980)

**Table 2:** Rate coefficients of radiative and non-radiative deactivations (*k_r_* and ***k_nr_***) of PhSt in PEG solvents, calculated from the fluorescence quantum yields (*Φ_F_*) and fluorescence decay times (*τ*_F_).

Solvent	*η*/ cP	*Φ_F_*	*τ*_F_ / ps	*k_r_* / 10^8^ s^−1^	*k_nr_* / 10^9^ s^−1^
PEG-200	64.0	0.0695	127	1.58	2.11
PEG-300	92.6	0.0919	441	1.64	1.62
PEG-400	123.3	0.101	561	1.59	1.41
PEG-600	178.3	0.133	637	1.79	1.17
90% PEG-600 10% PEG-1000	192.3	0.155	744	2.00	1.09

## Supplementary Materials

The following are available online. Description of the synthesis of PhSt, Description of the determination of the binding constants, Figure S1: Absorption and fluorescence spectra of PhSt in solvents of different polarities, Figure S2: Absorption spectra of PhSt in PEG solvents, Figure S3: Variation of the absorption spectra of PhSt with pH, Figure S4: Variation of the absorption spectra of a PhSt-CB7 mixture with pH, Figure S5: Job plot of the absorbance changes of PhSt-CB7 systems, Figure S6: 1H-NMR spectra of PhSt, CB7 and their mixtures, Figure S7: 1H-1H COSY NMR spectra of PhSt, a PhSt -CB7 1:1 mixture and a PhSt-Me_4_CB6 1:1 mixture, Figure S8: Theoretically calculated structures and stabilization energies of PhSt-cucurbituril complexes, Figure S9: Fluorescence spectra of a PhSt-CB7 assay in the presence of Lys, LysMe, LysMe2 and LysMe3 in different concentrations.
