# Supplementary material for: Binding Modes of a Phenylpyridinium Styryl Fluorescent Dye with Cucurbiturils
Source: Molecules. 2020 Nov 3;25(21):5111. doi: 10.3390/molecules25215111 (PMC7663148; doi:10.3390/molecules25215111)
Supplement: Supplementary file 1 [file molecules-25-05111-s001.pdf]

Synthesis of 4-(4-(dimethylamino)styryl)-1-phenylpyridinium iodide (PhSt)

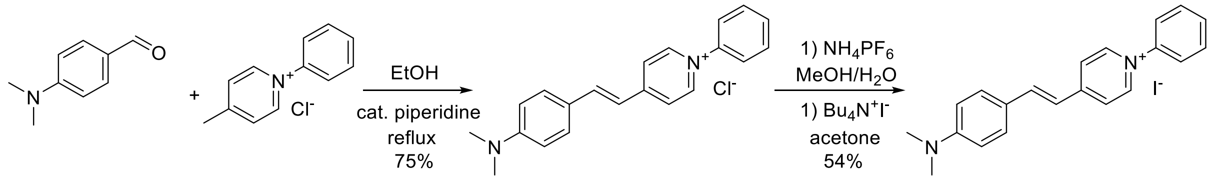

Scheme S1. Synthesis of PhSt.

First, the chloride salt of PhSt cation was prepared, by the Knoevenagel condensation of 4-dimethylaminobenzaldehyde and *N*-phenylpicolinium chloride.[1,2] To a solution of the chloride salt (0.51 g, 1.50 mmol) in 50% MeOH-water (10 mL) was added ammonium hexafluorophosphate (0.73 g, 4.5 mmol, 3 eq.) in 3 ml water. The resulting precipitate was filtered and washed thoroughly with water and water-methanol mixtures. The PF_6^- salt was dried in vacuo to obtain 0.47 g red solid (1.05 mmol). This was dissolved in 10 mL acetone and to this solution was added tetra-*n*-butylammonium iodide (1.1 g, 3 mmol, 3 eq.) in 2 mL acetonitrile and 5 ml acetone mixture. The resulting precipitate was filtered after several hours, washed with acetone multiple times and dried to obtain 0.35 g purple solid (54% for 2 steps).

1. (ref. 24 in the main article) Coe, B. J.; Harris, J. A.; Asselberghs, I.; Clays, K.; Olbrechts, G.; Persoons, A.; Hupp, J. T.; Johnson, R. C.; Coles, S.; Hursthouse, M. B.; Nakatani, K. Quadratic Nonlinear Optical Properties of N-Aryl Stilbazolium Dyes. Adv. Funct. Mater. 2012, 12, 110-116.

2. (ref. 27 in the main article) Bojtár, M.; Szakács, Z.; Hessz, D.; Bazsó, F. L.; Kállay, M.; Kubinyi, M.; Bitter, I. Supramolecular FRET modulation by pseudorotaxane formation of a ditopic stilbazolium dye and carboxylato-pillar[5]arene. Dyes Pigm. 2016, 133, 415-423.

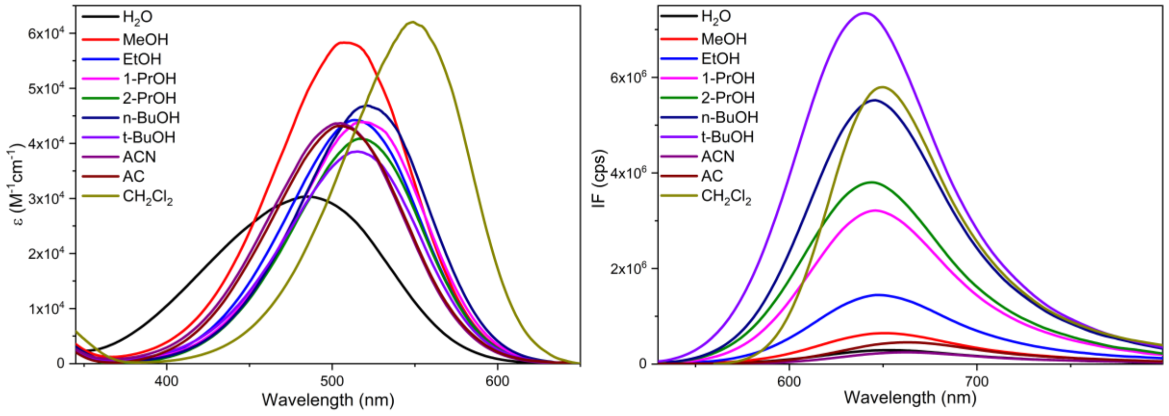

Figure S1. Absorption and fluorescence spectra of PhSt in solvents of different polarities.

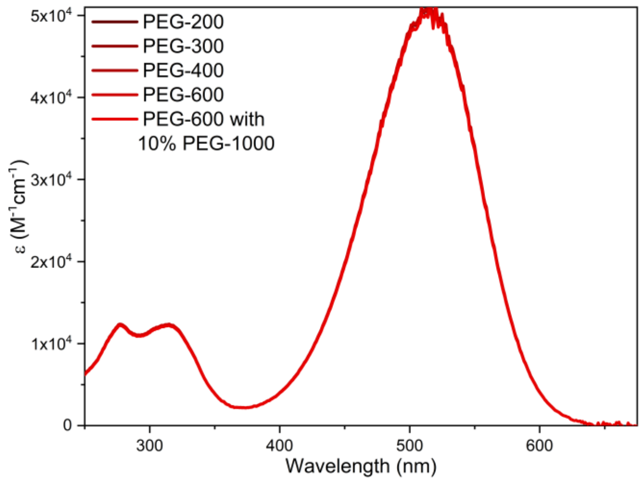

Figure S2. Absorption spectra of PhSt in PEG solvents.

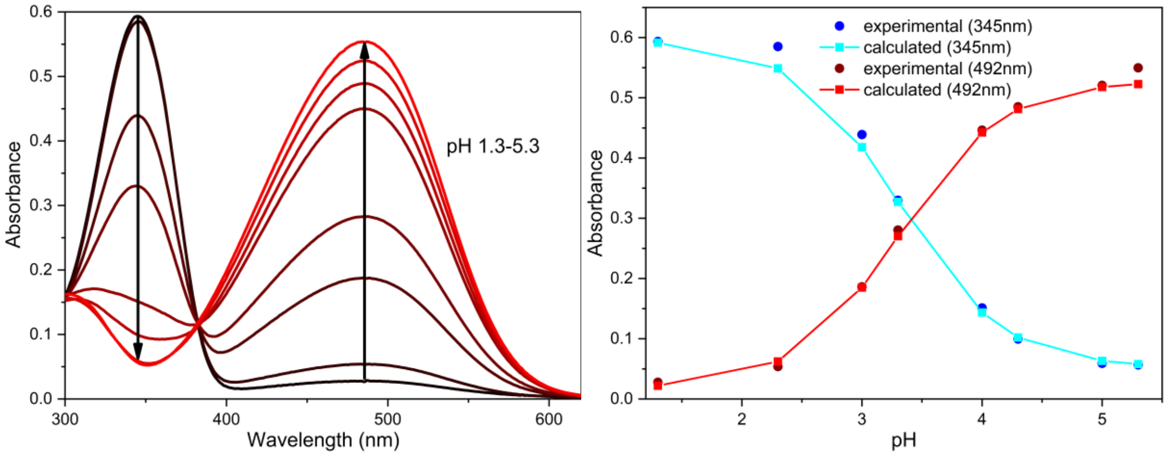

Figure S3. (**left**) The variation of the absorption spectra of PhSt in the 1.3–5.3 pH range; (**right**) the absorption coefficients at the band maxima, calculated with pKa = 3.32 at different pH-s.

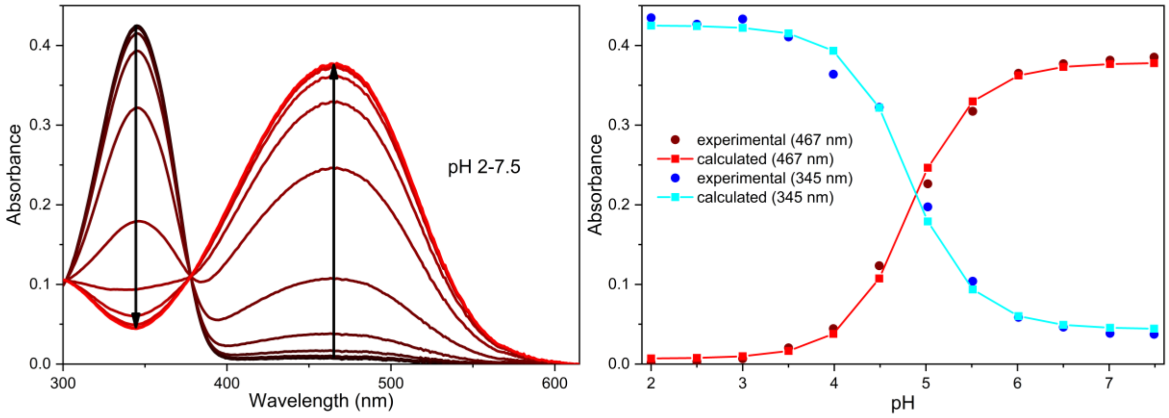

Figure S4. (**left**) The variation the absorption spectra of a PhSt – CB7 mixture in the 2.0–7.5 pH range. $[PhSt]_0 = 1.5 \times 10^{-5}$ M, $[CB7]_0 = 2.0 \times 10^{-5}$ M. (**right**) The absorbance values calculated with pKa = 5.08 at different pH-s.

Determination of the binding constants

The binding constants for the dye (D) – cucurbituril (CB) complexes

$$K_a = \frac{[D \cdot CB]}{[D][CB]} \tag{S1}$$

were calculated by a least square fitting to the fluorescence spectra of mixtures with the same initial dye concentration and different initial CB concentrations. The equilibrium concentrations in the individual samples were expressed in terms of K_a, $[D]_0$ and $[CB]_0$ from Equation (S1) and the molar balances

$$[D]_0 = [D] + [D \cdot CB] \tag{S2}$$

and

$$[CB]_0 = [CB] + [D \cdot CB] \tag{S3}$$

and substituted into the sum of squared residuals

$$S_{fl}^{tot} = \Sigma_i \Sigma_j \left(I_{i,j} - [D]_j \varphi_i^D - [D \cdot CB]_j \varphi_i^{D \cdot CB} \right)^2 \tag{S4}$$

$I_{i,j}$ in Equation (S4) is the fluorescence intensity of the j-th sample at the i-th wavelength, φ_i^{D} and $\varphi_i^{\mathrm{D \cdot CB}}$ are the relative fluorescence intensities of the respective species. A minimization of S_{fl}^{tot} using K_a and $\varphi_i^{\mathrm{D \cdot CB}}$ as fitting parameters yielded the value of the binding constant and also the spectrum of the complex.

Using an analogous algorithm, the binding constant of PhSt·CB7 was also calculated from the absorption spectra of PhSt-CB7 mixtures. In pH 8.0 buffers, a value of $K_a = 3.8\ 10^6\ M^{-1}$ was obtained which is in a reasonable agreement with the value of $K_a = 3.0 \times 10^6\ M^{-1}$ calculated from the fluorescence spectra. The complexation of PhSt by Me_4CB6 resulted only in minor changes of the absorption spectrum, not allowing a reliable determination of K_a.

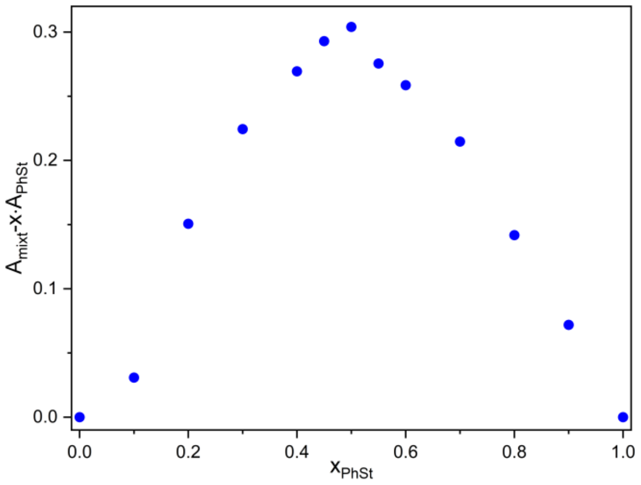

Figure S5. Job plot of the absorbance changes of PhSt-CB7 systems at 520 nm. Sum of the initial concentrations $[PhSt]_0 + [CB7]_0 = 4 \times 10^{-4}$ M.

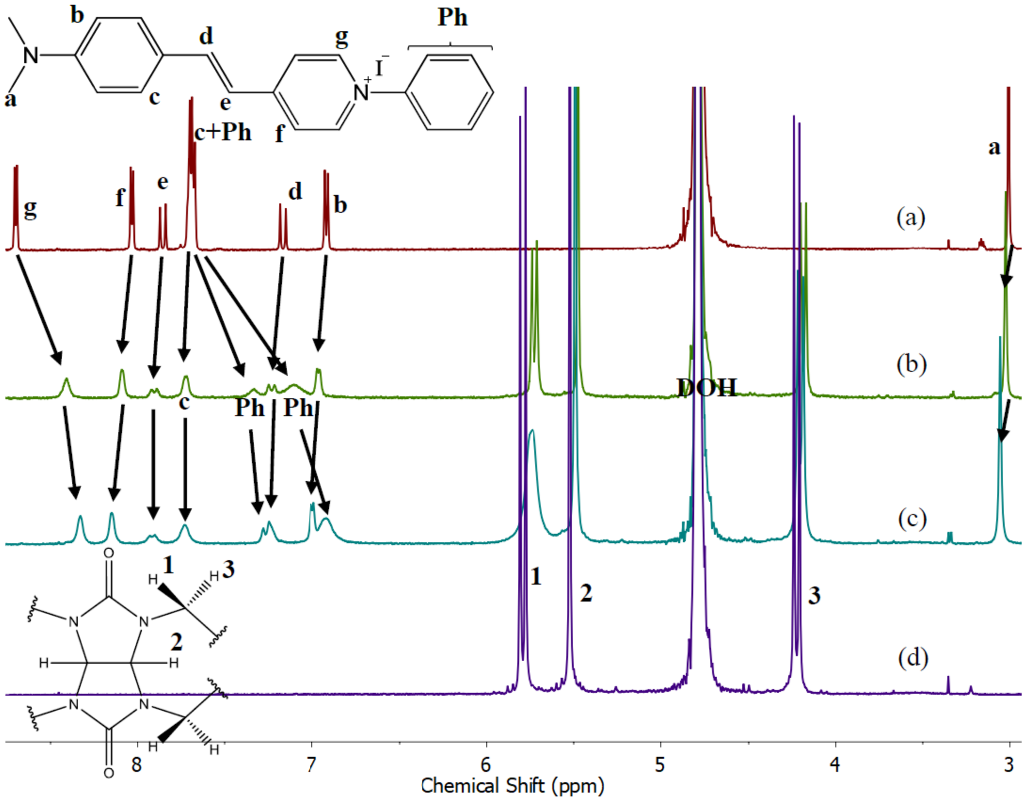

Figure S6. ^{1}H-NMR spectra (500 MHz, D_2O) of PhSt, CB7 and their mixtures. Concentrations (**a**) [PhSt] = 5 × 10^{-4} M; (**b**) [PhSt] = 5 × 10^{-4} M, [CB7] = 2.5 × 10^{-4} M; (**c**) [PhSt] = 5 × 10^{-4} M, [CB7] = 5 × 10^{-4} M; (**d**) [PhSt] = 5 × 10^{-4} M.

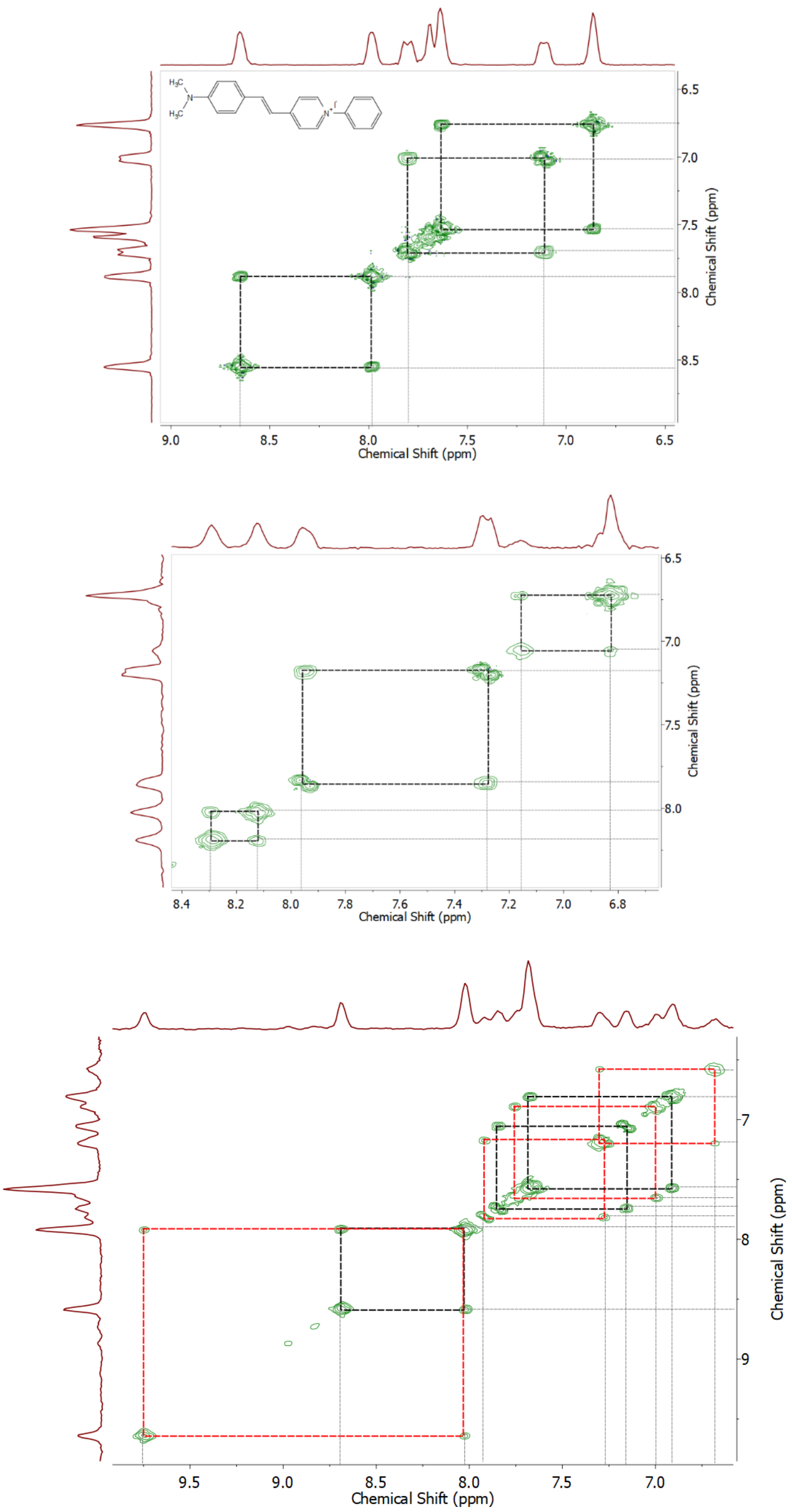

Figure S7. Range of aromatic protons in the ^{1}H-^{1}H COSY NMR spectra (500 MHz, D_2O) of (**top**) PhSt; (**middle**) PhSt-CB7 1:1 mixture; (**bottom**) PhSt-Me_4CB6 1:1 mixture; [PhSt] = [CB7] = [Me_4CB6] = 1.0 × 10^{-3} M.

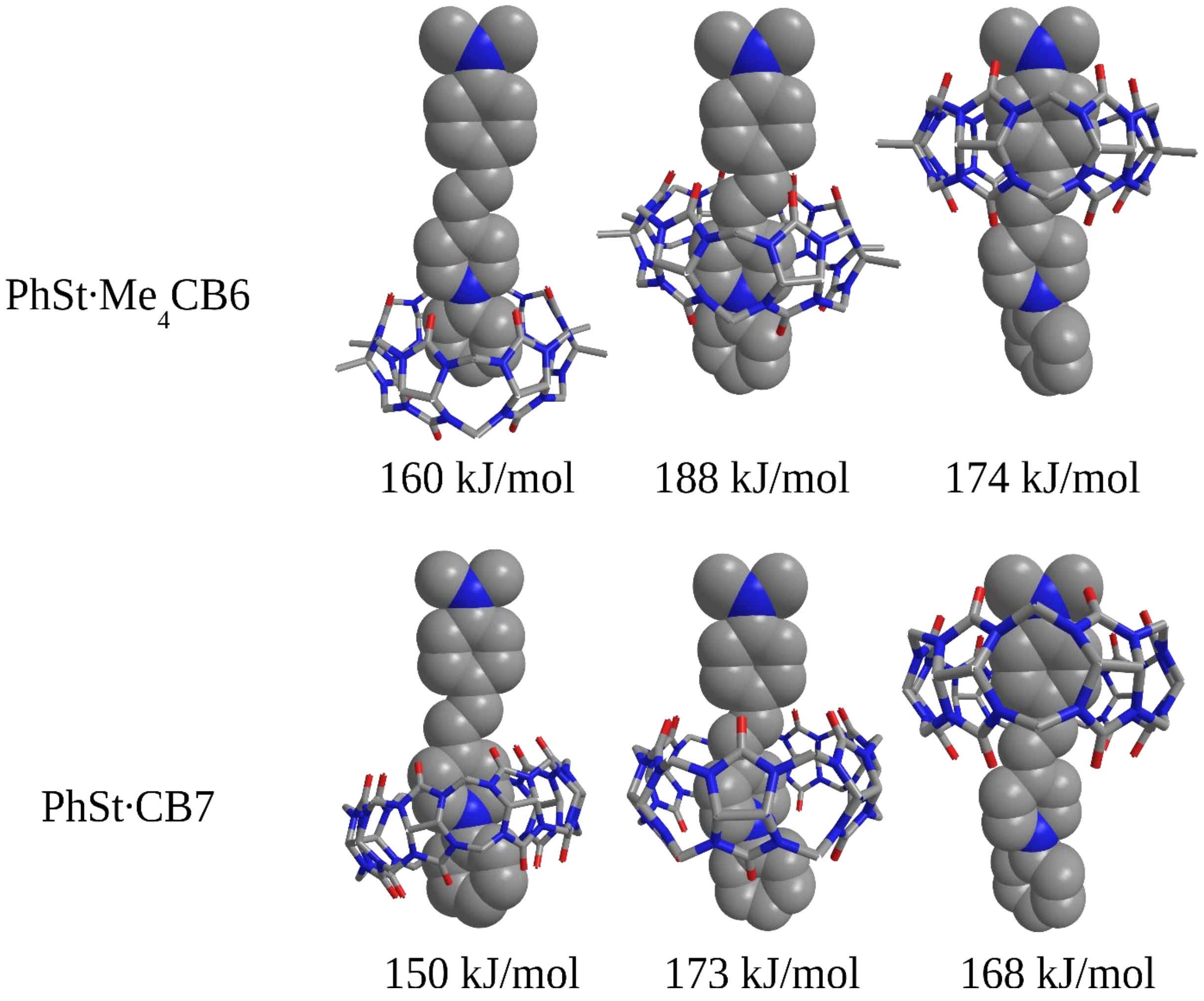

Figure S8. Theoretically calculated structures and stabilization energies of PhSt-cucurbituril complexes. Stabilization energies are defined as $E_{st} = -(E_{complex} - E_{host} - E_{guest})$.

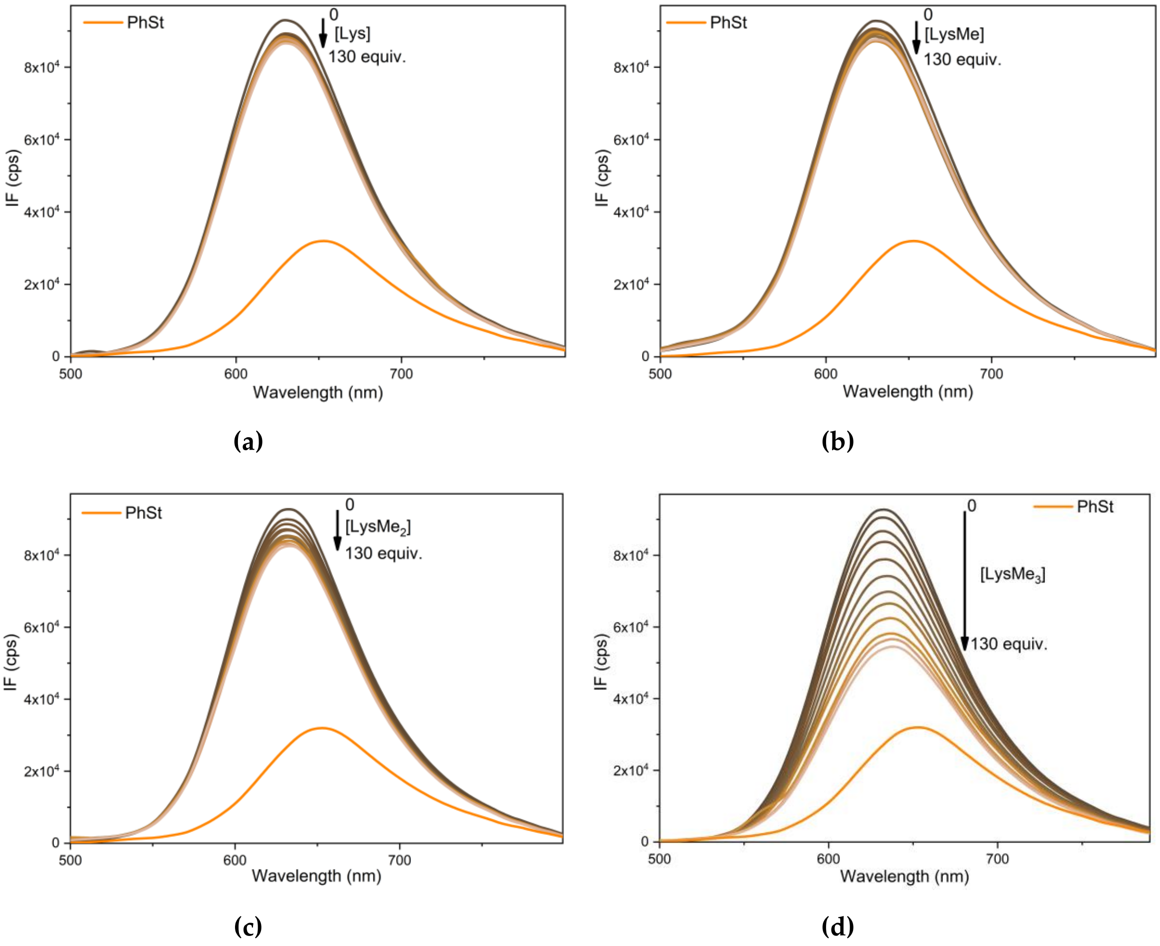

Figure S9. Fluorescence spectra of a PhSt-CB7 assay ($[PhSt]_0 = [CB7]_0 = 10^{-6}$ M) in the presence of 0 – 130 equiv. (**a**) Lys, (**b**) LysMe, (**c**) $LysMe_2$, (**d**) $LysMe_3$.
